# Impact of low-voltage area burden and topological obstacles on atrial fibrillation recurrence after pulmonary vein antrum isolation

**DOI:** 10.3389/fnetp.2026.1945351

**Published:** 2026-10-21

**Authors:** JungMin Choi, Young-Hae Go, Soonil Kwon, Kyung-Yeon Lee, Hyo-Jeong Ahn, So-Ryoung Lee, Taehun Kim, Jung Chan Lee, Eue-Keun Choi, Seil Oh

**Affiliations:** 1 Department of Internal Medicine, Seoul National University Hospital, Seoul, Republic of Korea; 2 Department of Internal Medicine, Cha University Bundang Medical Center, Seongnam, Republic of Korea; 3 Division of Cardiology, Department of Internal Medicine, Korea University Guro Hospital, Seoul, Republic of Korea; 4 Department of Internal Medicine, Seoul National University College of Medicine, Seoul, Republic of Korea; 5 Interdisciplinary Program in Bioengineering, Graduate School, Seoul National University, Seoul, Republic of Korea; 6 Department of Biomedical Engineering, Seoul National University College of Medicine, Seoul, Republic of Korea

**Keywords:** atrial fibrillation, electrophysiological parameters, low-voltage area, radiofrequency ablation, recurrence, topology, network physiology

## Abstract

**Background:**

Topological obstacles (TOs), electrically isolated regions such as the mitral annulus and atrial scars, can provide reentry circuits in the left atrium. Their quantity and relationship with low-voltage area (LVA) burden may influence recurrent atrial fibrillation (AF), but this impact remains poorly understood. We aimed to investigate the association between TO count, LVA burden, and post-ablation AF recurrence in patients undergoing *de novo* AF ablation.

**Methods:**

Patients were categorized by TO counts, identified using pulmonary vein antrum isolation lines and voltage maps. LVA burden (<0.5 mV) was quantified and grouped into quartiles. One-year AF/atrial tachycardia (AT) recurrence was evaluated across groups.

**Results:**

Among 237 patients (mean age 62.4 ± 8.8 years; 27.0% female, paroxysmal AF 42.6%, follow-up 1.0 ± 0. years), the majority had an intermediate TO count (TO = 3, 75.9%). Patients with the lowest TO count (TO = 1, 5.5%) were significantly older, had higher CHA_2_DS_2_-VASc scores, worse echocardiographic parameters, and the highest mean LVA burden (36.6% ± 24.8%). AF/AT recurrence occurred in 35.4% of patients. Although recurrence rates were numerically higher in patients with extreme TO counts (TO = 1 or ≥4) and lower in those with intermediate counts (TO = 2 or 3), these differences were not statistically significant (*p* = 0.433), and the TO group remained non-significantly associated with recurrence after multivariable adjustment for age, AF clinical type, left atrial diameter, left ventricular ejection fraction, CHA_2_DS_2_-VASc score, and LVA burden. TO counts differed significantly across LVA burden quartiles (*p* < 0.001), with the greatest variation observed in the group with the highest LVA burden. Dominant frequency and organization index showed no significant intergroup differences.

**Conclusion:**

Patients with extreme TO counts (TO = 1 or ≥4) showed a numerically higher, although not statistically significant, trend toward post-ablation recurrence compared with those with intermediate counts. TO quantity varied significantly with LVA burden, suggesting that atrial substrate heterogeneity may influence AF recurrence patterns.

## Introduction

Atrial fibrillation (AF) is the most common sustained cardiac arrhythmia, associated with significant morbidity and mortality, including risks of stroke, heart failure, and reduced quality of life (Tanaka et al., 2021; Linz et al., 2024; Ko et al., 2025). Pulmonary vein antrum isolation (PVAI) is a cornerstone treatment for AF (January et al., 2019; Van Gelder et al., 2024). However, recurrence rates remain high, especially in patients with advanced atrial remodeling or comorbidities (Kisheva and Yotov, 2021; Van Gelder et al., 2024). Structural remodeling, such as atrial fibrosis, can be assessed using low-voltage areas (LVAs) as a surrogate marker and has been linked to AF recurrence (Vlachos et al., 2017; Van Gelder et al., 2024). Additionally, electrophysiological metrics, such as dominant frequency (DF) and organization index (OI), have been explored to characterize AF substrate and predict outcomes after ablation (Jarman et al., 2012; Buttu et al., 2016; Choi et al., 2020; Pithon et al., 2021).

Despite the recognized importance of atrial substrate, ablation strategies beyond PVAI have shown inconsistent results. MRI-guided fibrosis ablation failed to improve outcomes (DECAAF II), and empiric linear lesions proved ineffective (STAR AF II) (Verma et al., 2015; Marrouche et al., 2022). Voltage-guided substrate modification yielded heterogeneous findings, with potential benefit in selected cohorts (ERASE-AF) but neutral results in others (VOLCANO, STABLE-SR II) (Masuda et al., 2020; Huo et al., 2022; Yang et al., 2022). These discrepancies suggest that targeting the substrate based solely on burden or presence may be insufficient; the spatial arrangement and functional impact of substrate abnormalities on atrial conduction networks remain poorly understood.

The left atrium (LA) contains intrinsic anatomical obstacles, primarily the mitral annulus and the pulmonary veins (Ho et al., 2012; Whiteman et al., 2019). LVAs, when sufficiently large, may also function as anatomical obstacles by creating regions of altered conduction (Love et al., 2001; Schaeffer et al., 2019). These structural features can further be conceptualized as topological obstacles (TOs), mathematical constructs reflecting the atrial geometry’s influence on arrhythmia dynamics. This topological framework was previously proposed by Duytschaever et al. (2024) as a unifying approach for understanding typical and atypical atrial flutter. While their work established the conceptual basis for the topological analysis of macroreentrant atrial tachycardia, its application to voltage-guided substrate characterization and AF (rather than atrial flutter) recurrence after PVAI has not been previously investigated. Understanding the interplay between TOs, LVA burden, and electrophysiological parameters could provide insights into optimizing patient selection and improving long-term outcomes after PVAI.

This study aimed to topologically analyze anatomical obstacles in the LA and evaluate their association with LVAs and recurrence patterns.

## Materials and methods

### Ethics statement

This study was conducted in accordance with the principles of the *Declaration of Helsinki*. The Institutional Review Board of the Seoul National University Hospital approved the study protocol (H-2503–021–1618). Written informed consent was waived due to the retrospective nature of the study and minimal risk to study participants.

### Study population

The study enrollment process is illustrated in Figure 1. Among 336 patients who underwent atrial fibrillation radiofrequency catheter ablation at Seoul National University Hospital between January 2023 and January 2024, the following exclusion criteria were applied: prior ablation history (***n*** = 78), absence of a complete LA voltage map (***n*** = 13), lack of post-ablation follow-up (***n*** = 4), and recurrence due to typical atrial flutter (AFL, ***n*** = 4). Patients with typical AFL recurrence were excluded because this arrhythmia arises from a distinct, well-defined macroreentrant circuit around the cavotricuspid isthmus, a mechanism fundamentally different from the LVA/TO-related substrate abnormalities investigated in this study. A total of 237 patients were included in the final analysis with up to 12-month follow-up.

**FIGURE 1:**
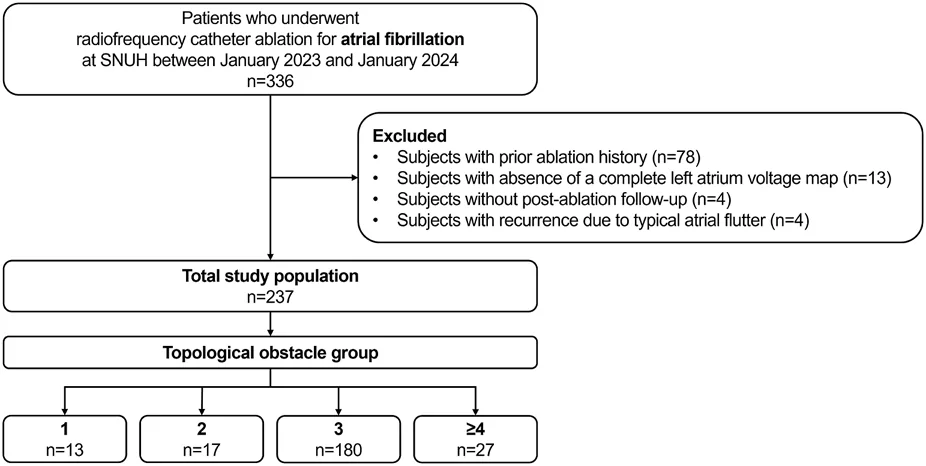
Study flow. A total of 336 patients undergoing radiofrequency catheter ablation for atrial fibrillation were screened, with 237 enrolled after excluding those with prior ablation, incomplete voltage maps, or lack of follow-up. The final study population was stratified based on the total number of topological obstacles (TOs) identified per patient. Subgroups 1 through 4 represent specific TO counts.

### Ablation procedure and low-voltage area estimation

Radiofrequency catheter ablation of PVAI was performed under deep conscious sedation using an electroanatomical mapping system (CARTO 3) with VISITAG SURPOINT software (Biosense Webster Inc., Diamond Bar, CA, United States). LA mapping and ablation were performed using either a Pentaray or Octaray catheter for mapping, and a Thermocool SmartTouch SF or QDOT MICRO catheter for ablation (all Biosense Webster Inc., CA, United States). Radiofrequency energy was delivered with differential settings based on anatomical location: 40 W (with an irrigation flow of up to 15 mL/min) for anterior and roof segments, and 30 W (with an irrigation flow of up to 8 mL/min) for the posterior, inferior, and carina segments. The target ablation index was set at ≥450 for anterior and roof segments, and ≥350 for the posterior, inferior, and carina segments to ensure optimal lesion formation (Lee et al., 2023). The standard ablation strategy consisted of PVAI. Additional lesions in the left or right atrium were performed at the discretion of the operator. LVA was identified through voltage mapping using high-density electroanatomic maps prior to ablation. Bipolar thresholds of <0.5 mV in sinus rhythm and <0.2 mV in AF were used in identifying LVA (Sanders et al., 2004; Takahashi et al., 2018; Starek et al., 2023). LVA burden was defined as the percentage of the total LA surface area occupied by regions meeting this voltage threshold, calculated as (LVA area/total LA surface area) × 100 using the CARTO 3 surface area measurement tool. To account for potential variations in voltage definitions based on rhythm, the mapping rhythm was recorded for all cases; 166 maps (70.0%) were acquired during sinus rhythm or pacing, while 71 maps (30.0%) were acquired during AF.

### Topological obstacles

Topology refers to the properties of geometric structures that are preserved under continuous deformation, such as stretching without tearing. In this study, the LA was modeled as a closed 2D surface containing TOs, defined as non-conductive regions that block electrical propagation. The identification of TOs was performed using a stepwise algorithm based on the pre-ablation voltage map. Initially, the left pulmonary vein antrum, the right pulmonary vein antrum, and the mitral valve were designated as the three fundamental TOs. These baseline obstacles were adjusted based on the presence of LVA or scar tissue. Discrete LVAs of sufficient size were classified as additional independent TOs, increasing the total count. Conversely, extensive scarring connecting anatomical structures resulted in the merging of obstacles, thereby reducing the total number of TOs (e.g., a scar bridging the left pulmonary veins and the right pulmonary veins creates a single, continuous obstacle) (Supplementary Figure S1). Finally, the topology was finalized by incorporating the specific ablation lines delivered during the procedure.

To differentiate functionally significant obstacles from negligible low-voltage spots, we determined the minimum size required to sustain a macroreentrant circuit. This was based on the principle that the path length around an obstacle must exceed the theoretical arrhythmia wavelength (λ).

Using the formula λ = action potential duration (APD) × conduction velocity (CV) and assuming a minimum atrial APD of 150 ms and a CV of 0.5 m/s, the minimum wavelength was calculated as follows:

0.150 s×0.5 m/s=0.075 m=75 mm.

These values were chosen to reflect diseased, fibrotic atrial tissue rather than normal myocardium: human atrial APD is shortened in chronic AF (approximately 140–330 ms, mean ≈ 216 ms), and left atrial conduction velocity in AF patients has been reported to range from 0.48 m/s to 0.77 m/s depending on fibrosis burden (Sánchez et al., 2014; Krul et al., 2015). Assuming a circular geometry (C = 2πr), a circumference of 75 mm corresponds to a radius of approximately 11.9 mm. Consequently, the minimum obstacle area required to support macroreentry was calculated as 4.45 cm^2^. For the purpose of this study, a rounded threshold of 4.5 cm^2^ was adopted, and LVAs smaller than this limit were not classified as TOs.

### Follow-up and outcomes

The primary outcome during the 12-month follow-up period was the development of AF or atrial tachycardia (AT) after 3 months post-ablation (blanking period). Recurrence was defined as any atrial tachyarrhythmia lasting ≥30 s, as documented by a 12-lead electrocardiogram (ECG) or Holter monitoring.

### Electrocardiographic signal processing of AF

In patients with recurrent AF, electrocardiographic signals were obtained from the 12-lead ECG recorded at the time of recurrence. Among the 12 electrical signals, lead V1 signals were used for analysis. NeuroKit2 Library with Python 3.11 was used to preprocess the ECG signals. QRS and T waves were separated using NeuroKit2 (Makowski et al., 2021). After segments of f-waves were extracted, each segment was detrended and concatenated. To calculate f-wave amplitude, the local maxima and minima were identified, and each maximum and minimum was then interpolated. Finally, two envelopes were generated, the difference between them was computed, and the mean of that difference was calculated. This value represented the f-wave mean amplitude (Squara et al., 2022). To find the DF and OI, the f-waves were down-sampled to 50 Hz. Using a fast Fourier transform (FFT) with a Hamming window, the DF had the largest amplitude. Harmonics of the DF were identified, and three frequencies, including the DF, were selected. Then, the OI was calculated using 1-Hz windows. The sample entropy of the f-wave was calculated using m = 2 and γ = 0.2.

### Statistical methods

Continuous variables were expressed as the mean ± standard deviation, and categorical variables were presented as numbers and percentages. Between-group comparisons of baseline characteristics were conducted using a one-way analysis of variance for continuous variables, with chi-square tests used for categorical variables. The distribution of TOs and recurrence patterns were analyzed across these quartiles. DF, OI, and LVA burden were compared across TOs using the Kruskal–Wallis test due to non-normal distribution. A linear regression analysis was performed to analyze DF and OI based on LVA burden. Recurrence patterns across TO groups were compared using the chi-square test in two ways: first, the overall distribution of recurrence type (no recurrence, AF, or AT) was compared across TO groups; second, the proportion of patients with any recurrence (AF or AT) versus no recurrence was compared across TO groups. Because Group 5 (TO = 5) comprised only a single patient, it was combined with Group 4 (TO = 4) for all subsequent analyses and is hereafter referred to as Group ≥4. To evaluate whether TO count was independently associated with 1-year AF/AT recurrence, a multivariable logistic regression model was constructed with the TO group (categorical, Group 3 as reference) as the primary predictor, adjusting for age, AF clinical type, LA diameter, left ventricular ejection fraction (LVEF), CHA_2_DS_2_-VASc score, and LVA burden. Variance inflation factors were calculated to assess multicollinearity among the covariates.

Statistical significance was set at *p* < 0.05. Statistical analyses were performed using R Statistical Software (Version 4.3.3).

## Results

The final study population comprised 237 patients, who were subsequently categorized into four groups based on TO count (Figure 2): Group 1 (*n* = 13), Group 2 (*n* = 17), Group 3 (*n* = 180), and Group ≥4 (n = 27).

**FIGURE 2:**
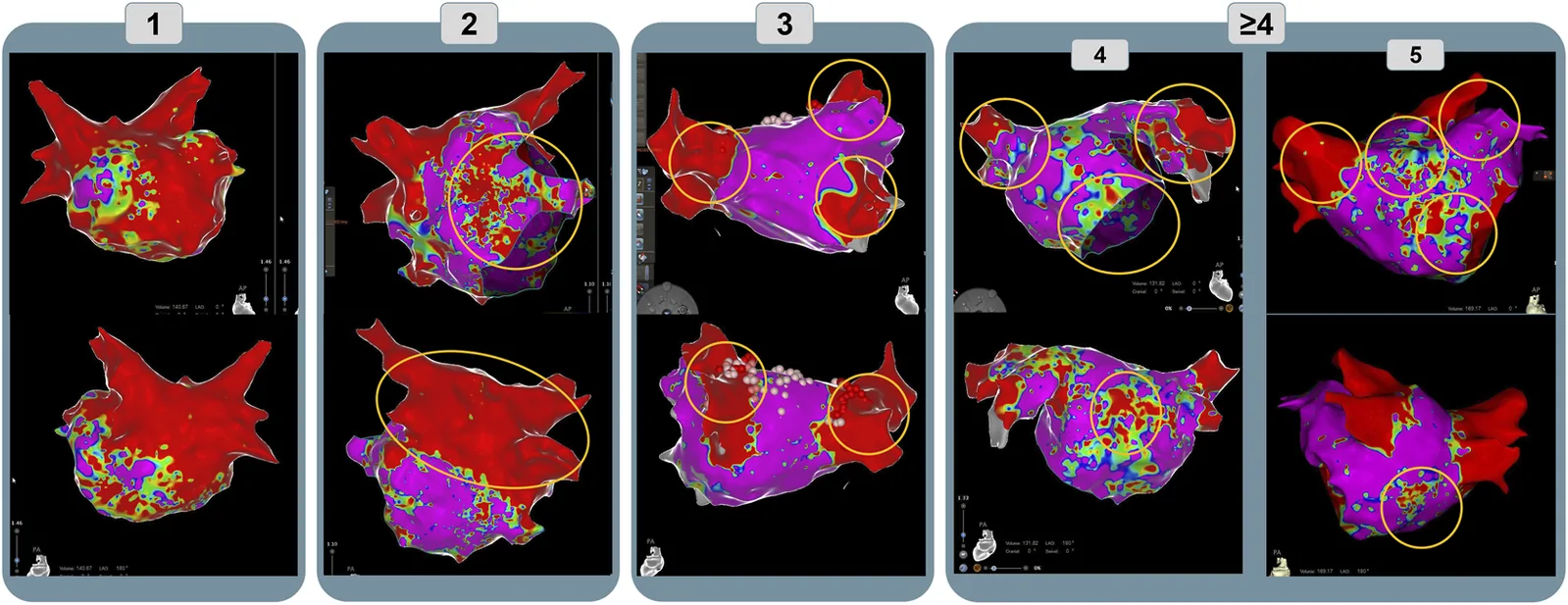
Representative examples of left atrium voltage maps in each topological obstacle group. Examples of voltage maps corresponding to each topological obstacle group (1–4) are displayed. The topological obstacles are highlighted with yellow circles.

### Baseline characteristics

Baseline characteristics stratified by TO group are presented in Table 1. The mean age of the study population was 62.4 ± 8.8 years (with a mean follow-up duration of 1.0 ± 0.1 years, 27.0% female). Subjects with a single TO (Group 1) tended to be older, have worse echocardiographic features (e.g., a lower left ventricular ejection fraction and a larger LA size), a higher CHA_2_DS_2_-VASc score, and a higher LVA burden. Among comorbidities, only valvular heart disease differed significantly across TO groups (*p* = 0.021). LVA burden ≥5% was present in all Group 1 patients (100.0%), compared with 24.9% of the overall population (*p* < 0.001). In addition to PVAI, adjunctive ablation was performed in 81 patients (34.1%), with the most common additional lesion being cavotricuspid isthmus ablation (*n* = 54, 22.8%).

**TABLE 1:** Baseline characteristics by topological obstacle group.

Characteristic	Total (n = 237)	Topological obstacle group				*p*-value
		1 (n = 13)	2 (n = 17)	3 (n = 180)	≥4 (n = 27)	
Age	62.4 ± 8.8	67.8 ± 6.0	65.4 ± 8.8	61.3 ± 9.0	64.9 ± 6.0	0.007
Sex (female)	64 (27.0%)	7 (53.8%)	7 (41.2%)	40 (22.2%)	10 (37.0%)	0.019
BMI	25.5 ± 3.3	22.9 ± 3.5	24.1 ± 2.8	25.7 ± 3.2	26.3 ± 3.3	0.003
Comorbidities						
Hypertension	126 (53.2%)	9 (69.2%)	8 (47.1%)	97 (53.9%)	12 (44.4%)	0.482
Diabetes	39 (16.5%)	4 (30.8%)	3 (17.6%)	24 (13.3%)	8 (29.6%)	0.084
Congestive heart failure	25 (10.5%)	3 (23.1%)	3 (17.6%)	17 (9.4%)	2 (7.4%)	0.310
Systemic embolism	19 (8.0%)	3 (23.1%)	1 (5.9%)	12 (6.7%)	3 (11.1%)	0.179
Coronary artery disease	18 (7.6%)	2 (15.4%)	1 (5.9%)	14 (7.8%)	1 (3.7%)	0.618
Valvular heart disease	12 (5.1%)	2 (15.4%)	3 (17.6%)	6 (3.3%)	1 (3.7%)	0.021
Cardiac surgery	4 (1.7%)	1 (7.7%)	0 (0.0%)	3 (1.7%)	0 (0.0%)	0.310
Social behavior						
Alcohol consumption*		​	​	​	​	0.950
No	115 (48.5%)	6 (46.2%)	8 (47.1%)	87 (48.3%)	14 (51.9%)	​
Yes	74 (31.2%)	3 (23.1%)	5 (29.4%)	57 (31.7%)	9 (33.3%)	​
Smoking	​	​	​	​	​	0.077
Current smoker†	23 (9.7%)	0 (0.0%)	3 (17.6%)	18 (10.0%)	2 (7.4%)	​
Ex-smoker	17 (7.2%)	3 (23.1%)	0 (0.0%)	9 (5.0%)	5 (18.5%)	​
Never	132 (55.7%)	6 (46.2%)	9 (52.9%)	102 (56.7%)	15 (55.6%)	​
AF						
AF clinical type	​	​	​	​	​	0.083
Paroxysmal	101 (42.6%)	2 (15.4%)	5 (29.4%)	86 (47.8%)	8 (29.6%)	​
Persistent	103 (43.5%)	7 (53.8%)	9 (52.9%)	71 (39.4%)	16 (59.3%)	​
Long-persistent	33 (13.9%)	4 (30.8%)	3 (17.6%)	23 (12.8%)	3 (11.1%)	​
CHA_2_DS_2_-VASc score	1.8 ± 1.3	3.1 ± 1.6	2.1 ± 1.3	1.6 ± 1.3	2.1 ± 1.1	<0.001
CHA_2_DS_2_-VASc score	1.5 ± 1.2	2.5 ± 1.5	1.6 ± 1.3	1.4 ± 1.2	1.7 ± 1.1	0.005
Echocardiographic measurements						
LVEF	57.2 ± 7.3	51.7 ± 12.6	59.4 ± 6.3	57.6 ± 6.5	55.7 ± 8.7	0.014
LA diameter	45.7 ± 7.0	50.1 ± 5.2	49.4 ± 6.1	44.6 ± 7.0	48.1 ± 6.7	0.001
LVA burden	5.6 ± 11.7	36.6 ± 24.8	18.0 ± 16.1	2.1 ± 3.5	6.6 ± 6.3	<0.001
LVA burden ≥5%	59 (24.9%)	13 (100.0%)	15 (88.2%)	19 (10.6%)	12 (44.4%)	<0.001

Categorical variables were presented as percentages, and continuous variables were presented as the mean and standard deviation.

Abbreviations: AF, atrial fibrillation; BMI, body mass index; DBP, diastolic blood pressure; LA, left atrium; LVA, low-voltage area; LVEF, left ventricular ejection fraction; SBP, systolic blood pressure.

* Data on alcohol consumption were unavailable for 48 patients (20.2%).

† Data on smoking status were unavailable for 65 patients (23.8%).

### Recurrence patterns and low-voltage area burdens across topological obstacle groups

Recurrence patterns varied across TO groups (Figure 3). Neither the overall distribution of recurrence rhythms (None vs. AF vs. AT) nor the comparison of any recurrence (AF or AT) versus no recurrence reached statistical significance (*p* = 0.584 and *p* = 0.433, respectively). Group 1 showed the highest rate of recurrence (46.2%), followed by Group ≥4 (44.4%), Group 3 (34.4%), and Group 2 (23.5%). AF was the predominant recurrence rhythm across all groups, with AT recurrence observed only sporadically (Groups 1, 2, 3, and ≥4: 0.0%, 25.0%, 6.4%, and 8.3%, respectively, among patients with recurrence). Among those patients with post-ablation AT, the mean cycle length was 235 ms. At the first post-ablation outpatient visit, amiodarone was the most common antiarrhythmic drug prescribed to patients who eventually developed recurrent AF, while flecainide was most frequently prescribed to those with subsequent AT recurrence (Supplementary Table S1). LVA burdens for each group are depicted in Supplementary Table S3. Group 1 demonstrated the highest LVA burden, whereas Group 3 showed the lowest (*p* < 0.001).

**FIGURE 3:**
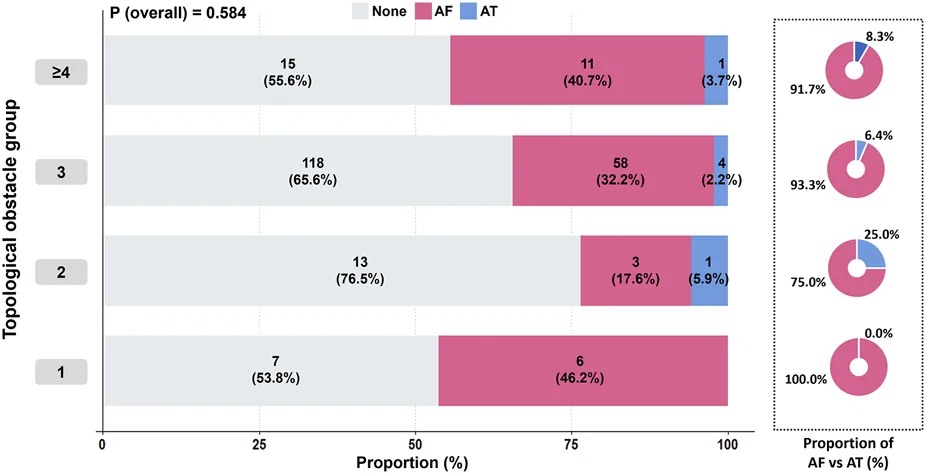
Recurrence patterns among the study population. The number and percentage of patients without recurrence, with atrial fibrillation (AF), and with atrial tachycardia (AT) are presented within each topological obstacle (TO) group on the left. The proportions of AF and AT among patients with recurrence are displayed as doughnut plots on the right. Patients with the extreme numbers of topological obstacles tended to experience recurrence more frequently, whereas those with intermediate burden showed a relatively low rate of recurrence, although these differences did not reach statistical significance. The overall distribution of recurrence types (None, AF, and AT) across the TO groups was analyzed using the chi-square test (P = 0.584). Abbreviations: AF, atrial fibrillation; AT, atrial tachycardia.

### Low-voltage area burden and topological obstacles

The distribution of patients across the LVA burden quartiles was as follows: Q1 (*n* = 60), Q2 (*n* = 60), Q3 (*n* = 58), and Q4 (*n* = 59). The boundaries of the LVA burden quartiles were as follows: Q1, 0%–0.35%; Q2, 0.35%–1.30%; Q3, 1.30%–4.88%; Q4, 4.88%–80.22%. Recurrence patterns and TO distribution for each quartile are summarized in Table 2, Supplementary Table S2, and Figure 4.

**TABLE 2:** LVA burden and recurrence pattern among LVA burden quartiles.

Variable	LVA burden (quartile)				*p*-value
	Q1 (*n* = 60)	Q2 (*n* = 60)	Q3 (*n* = 58)	Q4 (*n* = 59)	
LVA burden (%)	0.1 ± 0.1	0.8 ± 0.3	2.8 ± 1.0	18.9 ± 17.6	<0.001
Dominant frequency*	5.6 ± 0.6	5.9 ± 1.0	5.4 ± 1.4	5.1 ± 1.2	0.115
Organization index*	0.4 ± 0.1	0.4 ± 0.1	0.4 ± 0.1	0.5 ± 0.1	0.195
Recurrence pattern	​	​	​	​	0.018
No recurrence	48 (80.0%)	35 (58.3%)	38 (65.5%)	32 (54.2%)	​
AF/AT	12 (20.0%)	25 (41.7%)	20 (34.5%)	27 (45.8%)	​

Abbreviations: AF, atrial fibrillation; AT, atrial tachycardia; LVA, low-voltage area.

* n = 61 (DF and OI were calculated only from patients with acquired 12-lead AF ECG).

**FIGURE 4:**
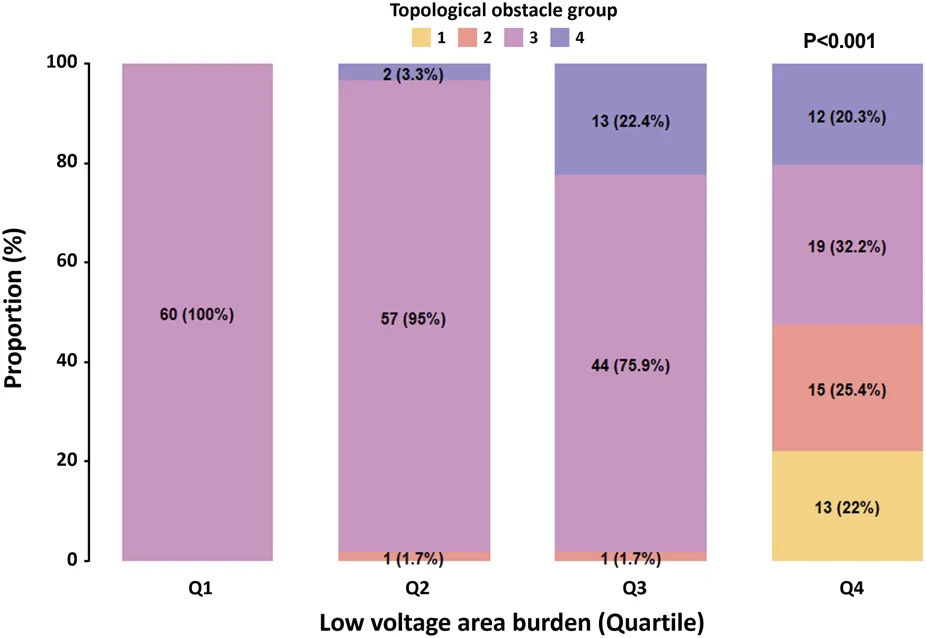
Proportional distribution of topological obstacles across low-voltage area burden quartiles. Patients were stratified into quartiles (Q1–Q4) based on the extent of low-voltage area (LVA) burden. A significant shift in topological configuration is observed as LVA burden increases. While a baseline of three topological obstacles (TOs) predominates in the lower quartiles, extensive scarring in Q4 is associated with a higher proportion of reduced TO counts.

AF recurrence was positively associated with an increase in LVA burden (Table 2). TO distribution varied significantly across quartiles (P < 0.001) (Figure 4). Q1 consisted exclusively of TO Group 3 (100%). Q2 and Q3 demonstrated progressively lower proportions of TO Group 3 and higher proportions of TO Group 4. Q4 was the only quartile to include patients with TO Group 1, together with Groups 2, 3, and ≥4.

When assessing recurrence patterns according to LVA burden quartiles, both any atrial tachyarrhythmia (AF or AT) and AF-only recurrence showed significant differences between the TO groups (both *p* < 0.001, Supplementary Table S2). TO Group 2 showed the lowest recurrence rate in every quartile in which it was represented. Because Q1 consisted exclusively of TO Group 3, a TO-based comparison within this quartile was not possible; in Q2 and Q3, recurrence rates did not increase consistently with higher TO groups, likely reflecting the small number of patients within each TO subgroup. Notably, AT recurrence exhibited a distinct pattern in relation to LVA burden. In the lower burden quartiles (Q1–Q3), AT recurrence was generally low (0.0%–4.5%), except for a high rate in TO Group ≥4 in Q2 (1/2, 50.0%), based on a very small sample. In Q4, AT recurrence was observed only among patients in TO Group 2 (6.6%), with no AT recurrence in TO groups 1, 3, or ≥4. However, statistical significance could not be tested due to the small sample size.

### Relationship between topological obstacles and dominant frequency, organization index, and low-voltage area burden

DF and OI measurements across TO groups are summarized in Supplementary Table S3 and Supplementary Figure S3. DF values in lead V1 ranged from 5.04 ± 0.87 (TO Group 1) to 5.66 ± 1.51 (TO Group ≥4). OI peaked in TO Group 1 (0.49 ± 0.09) and was lowest in TO Group 3 (0.45 ± 0.07). Neither the DF nor the OI differed significantly across the TO groups or the LVA burden quartiles (*p* = 0.727 and *p* = 0.629 for the TO groups; *p* = 0.115 and *p* = 0.195 for the LVA burden quartiles, respectively) (Table 2).

### Multivariable analysis of TO count and recurrence

To assess whether TO count was independently associated with 1-year AF/AT recurrence, a multivariable logistic regression model was constructed that included TO group (Group 3 as reference), age, AF clinical type, LA diameter, LVEF, CHA_2_DS_2_-VASc score, and LVA burden (Supplementary Table S4). After adjustment, none of the TO groups showed a statistically significant association with recurrence compared with Group 3 (Group 1: OR 0.46, 95% CI 0.08–2.72, *p* = 0.391; Group 2: OR 0.29, 95% CI 0.07–1.13, P = 0.075; Group ≥4: OR 1.17, 95% CI 0.49–2.79, *p* = 0.731; overall likelihood-ratio *p* = 0.194). None of the adjustment covariates reached statistical significance, either. The variance inflation factors for all covariates were <2, indicating no substantial multicollinearity between TO group and LVA burden.

## Discussion

In this cohort of AF patients undergoing *de novo* AF radiofrequency catheter ablation, TO stratification revealed distinct clinical and arrhythmic profiles. The main findings are as follows: (1) Patients with a single obstacle (Group 1) were older, had a higher CHA_2_DS_2_-VASc score, worse cardiac function, and an LVA burden of ≥5% in all patients. (2) Patients with extreme TO counts (groups 1 or ≥4) showed a numerically higher recurrence rate than those with intermediate TO counts (groups 2 or 3), although this difference was not statistically significant. (3) No significant intergroup difference was observed in DF or OI. (4) On multivariable analysis adjusting for established predictors of recurrence, the TO group remained non-significantly associated with AF/AT recurrence, consistent with the univariable result; the numerically higher recurrence observed in extreme TO groups should therefore be regarded as an exploratory, hypothesis-generating observation rather than a confirmed finding, warranting confirmation in larger, adequately powered cohorts. To our knowledge, this is the first study to apply the topological framework proposed by Duytschaever et al. (2024) to voltage mapping, examining its relationship with AF recurrence after PVAI, LVA burden, and electrophysiological parameters, such as DF and OI.

LVA in the LA is associated with a higher risk of atrial tachyarrhythmias following AF catheter ablation (Vlachos et al., 2017; Masuda et al., 2018; Starek et al., 2023; Georgi et al., 2024). These observations have prompted the concept of atrial substrate modification based on LVA, although outcomes from this approach remain controversial (Rolf et al., 2014; Huo et al., 2022; Junarta et al., 2022; Marrouche et al., 2022). The DECAAF II trial compared PVAI alone versus PVAI combined with additional ablation targeting fibrotic areas identified through MRI-delayed enhancement imaging, finding no significant benefit from scar homogenization in persistent AF patients (Marrouche et al., 2022). Conversely, another multicenter randomized clinical trial utilizing pre-ablation voltage mapping reported significantly improved outcomes in patients with persistent AF when PVAI was combined with individualized ablation targeting atrial low-voltage myocardium (Huo et al., 2022). Collectively, recent trials highlight scar burden as a critical determinant of ablation success. Our study provides additional observational insights into recurrence patterns in relation to counts and LVA burden, an aspect relatively underrepresented in previous research.

In this study, we found that an extreme number of TOs was numerically associated with a higher likelihood of AF recurrence following PVAI, whereas intermediate obstacle numbers showed a relatively low risk of recurrence; however, this association was not statistically significant in the univariable comparison and remained non-significant after multivariable adjustment. To interpret these observations, we hypothesize that both extremes of TO count may favor chaotic wave propagation, albeit through different substrate configurations. AF has previously been proposed to be maintained by multiple wandering wavelets, stable rotors (mother rotors), or, more recently, by multiple focal activation patterns (Jalife et al., 1998; Lee et al., 2020; Schotten et al., 2021). Within this framework, the two extreme groups represent opposite substrate configurations: TO = 1 reflects a single large, confluent area of scarring with a high LVA burden, whereas TO≥4 reflects multiple discrete, smaller low-voltage areas. Both substrate patterns might theoretically favor chaotic wave propagation, potentially explaining the numerically higher trend toward AF recurrence observed in these groups. Therefore, the differing recurrence patterns observed in our study may potentially be explained by the interplay between structural obstacles and electrophysiological conduction dynamics.

The pattern of AF/AT recurrence was not solely dependent on TO count or LVA burden. We found that even within the same LVA burden quartile, recurrence patterns differed by TO count. These findings highlight that the relationship between TO counts and recurrence is fundamentally influenced by the underlying atrial substrate. Although TO count is inherently derived from the voltage map and therefore associated with LVA burden, this relationship is not simply linear: patients with a single large, confluent obstacle (Group 1) exhibited the highest LVA burden, whereas the predominant intermediate group (Group 3) exhibited the lowest, reflecting the fact that TO count captures the spatial configuration and connectivity of scar tissue rather than its total extent. TO count is therefore not redundant with LVA burden but reflects an additional structural dimension of the atrial substrate. Accordingly, a combined assessment of TO counts and LVA burden is crucial for accurately predicting the risk of post-ablation AF recurrence.

DF and OI were assessed as independent, signal-derived measures of fibrillatory organization to test whether the atrial substrate heterogeneity captured by TO count and LVA burden was also reflected in global electrophysiological complexity during recurrent AF. Previous studies have shown that patients with persistent AF and larger LA dimensions typically exhibit lower DF and higher OI, which are linked to reduced AF thresholds (Takahashi et al., 2006; Tobón et al., 2012; Choi et al., 2020). In our study, however, DF and OI did not show a significant intergroup difference across either the TO groups or the LVA burden quartiles. Although DF varied across LVA burden quartiles, peaking in Q2 and declining thereafter, it did not reach statistical significance. This discrepancy may be attributed to several factors, including a limited sample size and the complex relationship between TO groups and LVA burden. This lack of an association suggests that global, signal-derived organization metrics may not capture the same dimension of atrial substrate heterogeneity as structurally defined TO count and LVA burden, indicating that anatomical and electrophysiological substrate characterization may provide complementary rather than redundant information.

### Limitations

This study has several limitations. First, the retrospective design introduces potential selection bias and unmeasured confounders, including operator-dependent ablation strategies and undocumented comorbidities, which may have influenced the outcomes. Second, the single-center nature of this study and the small sample size in Group 1 (n = 13, 5.5%) limit the generalizability of the findings to broader populations. Third, the study’s reliance on LVA to delineate TOs constitutes a limitation, as the nature of LVA as a true surrogate for scar tissue is still under debate. Fourth, voltage measurements obtained via multipolar catheters are susceptible to variations in the underlying rhythm, wavefront directionality, and electrode configuration. As these factors were not fully controlled, they represent potential confounders that may affect the generalizability of our results. Fifth, the APD and CV values used to derive the TO size threshold were fixed, literature-derived approximations rather than patient-specific measurements; these parameters are known to vary regionally and temporally within the atrium and are influenced by scar geometry, autonomic tone, and AF duration, which may affect the precision of the TO classification threshold. The sensitivity of our findings to this threshold was not formally tested against alternative cutoff values in the present retrospective cohort; future studies incorporating such sensitivity analyses are warranted. Sixth, the assumption of circular obstacle geometry may under-count small, irregularly shaped LVAs whose actual boundary length exceeds the theoretical wavelength despite a sub-threshold area; a supplementary sensitivity analysis suggested this effect is modest for mildly elongated shapes but more pronounced for markedly elongated ones. A convex hull-based boundary length criterion would remove this shape assumption and represent a direction for future methodological refinement. These limitations highlight the need for prospective, multicenter studies with larger cohorts and extended follow-up to validate these findings.

## Conclusion

Patients with extreme TO counts (groups 1 or ≥4) showed numerically higher post-ablation recurrence rates than those with intermediate counts, but this difference was not statistically significant in either univariable or multivariable analysis. The number of TOs varied significantly with the patient’s LVA burden, with the most diverse counts seen in patients with the highest LVA burden. These findings suggest that atrial substrate heterogeneity, reflected in both TO count and LVA burden, may influence the pattern of AF recurrence, warranting confirmation in larger prospective cohorts.

## Data Availability

The datasets presented in this study are not readily available because they contain patient‐level clinical information, and sharing is restricted by institutional policy and the conditions of Institutional Review Board approval (H‐2503‐021‐1618). Requests to access the datasets should be directed to the corresponding author.
