# Supplementary material for: Impact of low-voltage area burden and topological obstacles on atrial fibrillation recurrence after pulmonary vein antrum isolation
Source: Front Netw Physiol. 2026 Oct 21;6:1945351. doi: 10.3389/fnetp.2026.1945351 (PMC13730920; doi:10.3389/fnetp.2026.1945351)
Supplement: Supplementary file 1 [file Supplementaryfile1.docx]

**Supplemental Materials**

**Supplemental Tables**

**Table S1. Antiarrhythmic drug prescribed on first post-ablation among patients with atrial fibrillation or atrial tachycardia recurrence**

**Table S2. Number of recurrences according to low-voltage area burden and topological obstacle groups**

**Table S3. Comparison of dominant frequency, organization index, and LVA burden by topological obstacles among patients with 12 lead-ECG confirmed AF recurrence**

**Table S4.** **Multivariable logistic regression analysis of factors associated with 1-year AF/AT recurrence**

**Supplemental Figures**

**Figure S1. Schematic diagram of topological obstacles**

**Figure S2. Comparison of Low-Voltage Area Burden Among Topological Obstacle Groups**

**Figure S3. Trends of dominant frequency and organization index by topological obstacle group.**

**Supplemental Tables**

**Table S1. Antiarrhythmic drug prescribed on first post-ablation among patients with atrial fibrillation or atrial tachycardia recurrence**

|  | **AF**  **(*n*=78)** | **AT**  **(*n*=6)** | **P-value** |
| --- | --- | --- | --- |
| **Verapamil or Diltiazem** | 6 (7.7%) | 1 (16.7%) | 1.000 |
| **Propafenone** | 11 (14.1%) | 1 (16.7%) | 1.000 |
| **Flecainide** | 13 (16.7%) | 3 (50.0%) | 0.143 |
| **Pilsicainide** | 0 (0.0%) | 0 (0.0%) | - |
| **Amiodarone** | 37 (47.4%) | 1 (16.7%) | 0.301 |
| **Sotalol** | 0 (0.0%) | 0 (0.0%) | - |
| **Dronedarone** | 3 (3.8%) | 0 (0.0%) | 1.000 |

Abbreviation: AF, atrial fibrillation; AT, atrial tachycardia

**Table S2. Number of recurrences according to low-voltage area burden and topological obstacle groups**

| **TO group** | | **LVA burden (Quartile)** | | | | **P-value** |
| --- | --- | --- | --- | --- | --- | --- |
|  |  | **Q1**  **(*n*=60)** | **Q2**  **(*n*=60)** | **Q3**  **(*n*=58)** | **Q4**  **(*n*=59)** |  |
| **AF or AT (event/n; %)** | |  |  |  |  | <0.001 |
|  | **1** | - | - | - | 6/13 (46.1%) |  |
|  | **2** | - | 0/1 (0.0%) | 0/1 (0.0%) | 4/15 (26.6%) |  |
|  | **3** | 12/60 (20%) | 24/57 (42.1%) | 16/44 (36.3%) | 10/19 (52.6%) |  |
|  | **≥4** | - | 1/2 (50.0%) | 4/13 (30.7%) | 7/12 (58.3%) |  |
| **AF** |  |  |  |  |  | <0.001 |
|  | **1** | - | - | - | 6/13 (46.2%) |  |
|  | **2** | - | 0/1 (0.0%) | 0/1 (0.0%) | 3/15 (20.0%) |  |
|  | **3** | 12/60 (20%) | 22/57 (25.2%) | 14/44 (31.8%) | 10/19 (52.6%) |  |
|  | **≥4** | - | 0/2 (0.0%) | 4/12 (33.3%) | 7/12 (58.3%) |  |
| **AT** |  |  |  |  |  | N/A |
|  | **1** | - | - | - | 0/13 (0.0%) |  |
|  | **2** | - | 0/1 (0.0%) | 0/1 (0.0%) | 1/15 (6.6%) |  |
|  | **3** | 0/60 (0.0%) | 2/57 (3.5%) | 2/44 (4.5%) | 0/19 (0.0%) |  |
|  | **≥4** | - | 1/2 (50.0%) | 0/12 (0.0%) | 0/12 (0.0%) |  |

Abbreviation: AF, atrial fibrillation; AT, atrial tachycardia; LVA, low-voltage area; TO, topological obstacle

**Table S3. Comparison of dominant frequency, organization index, and LVA burden by topological obstacles among patients with 12 lead-ECG confirmed AF recurrence**

|  | **Topological obstacle group** | | | | **P-value** |
| --- | --- | --- | --- | --- | --- |
|  | **1**  **(*n*=6)** | **2**  **(*n*=3)** | **3**  **(*n*=43)** | **≥4**  **(*n*=9)** |  |
| **Dominant frequency** | 5.04 ± 0.87 | 5.07 ± 1.35 | 5.47 ± 1.14 | 5.66 ± 1.51 | 0.727 |
| **Organization index** | 0.49 ± 0.09 | 0.46 ± 0.01 | 0.45 ± 0.07 | 0.45 ± 0.09 | 0.629 |
| **LVA burden** | 46.81 ± 28.48 | 14.60 ± 6.09 | 3.21 ± 4.04 | 10.10 ± 5.11 | <0.001 |

Abbreviation: LVA, low-voltage area.

**Table S4. Multivariable logistic regression analysis of factors associated with 1-year AF/AT recurrence**

| **Variable** | **OR** | **95% CI** | **P-value** |
| --- | --- | --- | --- |
| **TO group (ref: Group 3)** |  |  |  |
| **Group 1** | 0.46 | 0.08–2.72 | 0.391 |
| **Group 2** | 0.29 | 0.07–1.13 | 0.075 |
| **Group ≥4** | 1.17 | 0.49–2.79 | 0.731 |
| **Age (per year)** | 0.99 | 0.95–1.03 | 0.528 |
| **AF clinical type (ref: Paroxysmal)** |  |  |  |
| **Persistent** | 1.54 | 0.80–2.97 | 0.193 |
| **Long-persistent** | 2.00 | 0.84–4.77 | 0.117 |
| **LA diameter (per mm)** | 1.03 | 0.99–1.08 | 0.188 |
| **LVEF (per %)** | 1.01 | 0.97–1.05 | 0.704 |
| **CHA_2_DS_2_-VASc score (per point)** | 1.09 | 0.83–1.45 | 0.536 |
| **LVA burden (per %)** | 1.03 | 0.99–1.07 | 0.142 |

Variance inflation factors for all covariates were <2, indicating no substantial multicollinearity.

Abbreviation: AF, atrial fibrillation; AT, atrial tachycardia; CI, confidence interval; LA, left atrium; LVA, low-voltage area; LVEF, left ventricle ejection fraction; OR, odds ratio; TO, topological obstacle.

**Supplemental Figures**

**Figure S1. Schematic diagram of topological obstacles**

**Figure S2. Comparison of Low-Voltage Area Burden Among Topological Obstacle Groups**

**Figure S3. Trends of dominant frequency and organization index by topological obstacle group.**
